# Supplementary material for: Marine or freshwater: the role of ornamental fish keeper’s preferences in the conservation of aquatic organisms in Brazil
Source: PeerJ. 2022 Nov 11;10:e14387. doi: 10.7717/peerj.14387 (PMC9661971; doi:10.7717/peerj.14387)
Supplement: Supplemental Information 2 [file peerj-10-14387-s002.docx]

**Marine or freshwater: the role of ornamental fish keeper's preferences in the conservation of aquatic organisms in Brazil**

**Supplementary material**

**Table 1A.** Questionnaire applied to hobbyists participating in fishkeeping groups on Facebook.

| **Questions** |
| --- |
| **Socioeconomic** |
| What city do you live in? |
| What is your gender? |
| How old are you? |
| What do you do for a living? |
| What is your education level? |
| What is your monthly income? |
| **About the hobby** |
| How long have you been doing this hobby? |
| What motivated you to choose fish as pets? |
| Do you own marine or freshwater fish? |
| Where did you gain experience in fishkeeping? |
| What do you consider most important when acquiring a new pet? |
| Where do you usually buy your fish? |
| How much on average do you spend on your hobby/aquarium maintenance? |
| How many fish do you currently have? |
| List the names of the fish you own. If you know the scientific names, please include them. |
| Do you have invertebrates in your aquarium? If yes, which ones? |
| What type of aquarium/tank do you have? |
| Have any of the animals you own been captured by you? If so, which animal? How was it captured? Where was it captured? |
| What is the food offered to your animals? |
| Is the aquarium/tank decorated? With which items? |
| What is the frequency of care (cleaning) of the aquarium/tank? |
| How often are partial water changes performed? |
| Have you ever managed to breed an animal? |
| If your fish ever reproduced, what did you do with the young? |
| Has any animal ever been sick? If so, what type of disease did you experience and what was the treatment? |
| When one of your animals gets sick, where do you seek treatment? |
| When you want to get rid of an animal, what fate do you give it? |
| Have you ever released fish into the wild? If yes, where and what was the fish? |
| Which of the following information about the future of fish farming/trading do you agree with? |
| More fish will be available for trade in the future due to cultivation. |
| Fewer fish will be available for trade in the future due to overfishing and loss of natural habitats. |

**Table 2A.** Answers from fishkeepers interviewed to questions about their relationship with the hobby.

| **Question** | **Frequency (n)** |
| --- | --- |
| How long have you been practicing the hobby? |  |
| Less than one year | 11% (104) |
| Between one and five years | 29% (268) |
| Between five and ten years | 14% (135) |
| More than ten years | 18% (164) |
| Since childhood | 26% (234) |
| What motivated you to choose fish as pets? |  |
| The beauty of aquariums and fish | 76% (790) |
| Encouragement from friends or family | 7% (72) |
| Quiet fish behavior | 6% (59) |
| commercial interest | 4% (43) |
| Passion for fish and aquariums | 3% (27) |
| Therapy for treating depression/anxiety | 2% (24) |
| Interest in fish ecology/biology | 1.5% (16) |
| How did you gain experience in fishkeeping? |  |
| Internet search (Google, blogs, Youtube) | 22% (703) |
| Specific forums for fishkeepers | 20% (618) |
| Consulting specialized aquarium sites | 17% (522) |
| By trial and error | 16% (505) |
| Consulting specific literature | 11% (340) |
| Aquarium groups on social networks (Facebook, Whatsapp) | 7% (225) |
| Professional advice | 3% (101) |
| Taking class | 3% (92) |
| What do you consider most important to acquire a new fish? |  |
| Behavior | 26% (537) |
| Aesthetics | 22% (441) |
| Provenance | 16% (338) |
| If it performs any specific function in the aquarium | 14% (291) |
| Price | 8% (161) |
| Ease of care | 8% (158) |
| Shopkeeper's recommendation | 3% (64) |
| Compatibility with the aquarium and fauna you already own | 63% (63) |
| Where do you usually acquire your fish? |  |
| Stores or petshops | 55% (776) |
| Buy directly from suppliers | 18% (255) |
| Fishkeeping groups on social networks | 15% (217) |
| Online stores | 9% (128) |
| Capture directly in nature | 3% (39) |

* Percentages were calculated based on the number of citations. Thus, the sum of n may exceed the total number of participants (n = 906) when the questions allowed more than one answer option.

**Table 3A.** Answers from Brazilian aquarists interviewed to questions related to hobby maintenance.

| **Question** | **Type of fish kept** | | | |
| --- | --- | --- | --- | --- |
|  | **Freshwater (%, n)** | **Marine (%, n)** | **Both types**  **(%, n)** | **General**  **(%, n)** |
| How many fish do you currently have? |  |  |  |  |
| Less than 5 fish | 10% (77) | 24% (11) | 9% (7) | 10% (95) |
| Between 5 and 10 fish | 19% (147) | 46% (21) | 19% (16) | 20% (184) |
| Between 10 and 20 fish | 26% (202) | 19% (9) | 16% (14) | 25% (225) |
| Between 20 and 50 fish | 39% (303) | 11% (5) | 50% (42) | 39% (350) |
| Between 50 and 100 fish | 1.5% (13) | 0 | 1% (1) | 1.5% (14) |
| Between 100 and 500 fish | 1.3% (10) | 0 | 3% (2) | 1.3% (12) |
| Between 500 and 1000 fish | 1.1% (9) | 0 | 0 | 1% (9) |
| More than a thousand | 1.1% (9) | 0 | 1% (1) | 1% (10) |
| How much do you spend monthly on the hobby? |  |  |  |  |
| Don't spend anything | 0.5% (4) | 0 | 0 | 0.5% (4) |
| Less than US$ 20 | 53% (437) | 24% (11) | 32% (27) | 52% (475) |
| Between US$20 and US$ 100 | 38% (293) | 63% (29) | 51% (43) | 40% (365) |
| Between US$100 and US$ 200 | 4% (34) | 8.7% (4) | 6% (5) | 5% (43) |
| Between US$200 and US$ 400 | 3.5% (3) | 0 | 0.5% (4) | 1% (7) |
| More than US$ 400 | 3.5% (3) | 2% (2) | 3.5% (3) | 1% (8) |
| Do not know | 2% (2) | 0 | 2% (2) | 0.5% (4) |
| What is the cleaning frequency? |  |  |  |  |
| Daily | 0.4% (3) | 2% (1) | 1% (1) | 1% (5) |
| More than once a week | 10% (77) | 22% (10) | 19% (16) | 11% (103) |
| Weekly | 40% (313) | 41% (19) | 38% (32) | 40% (364) |
| Biweekly | 28% (216) | 17% (8) | 30% (25) | 27% (249) |
| Monthly | 18% (141) | 15% (7) | 11% (9) | 17% (157) |
| Quarterly | 1% (7) | 0 | 0 | 1% (7) |
| Semiannual | 1% (7) | 0 | 0 | 1% (7) |
| As required | 0.6% (5) | 0 | 1% (1) | 1% (6) |
| Other answers | 1% (7) | 2% (1) | 0 | 1% (8) |
| What is the frequency of partial water changes? |  |  |  |  |
| Weekly | 38% (296) | 13% (6) | 33% (28) | 36% (330) |
| More than once a week | 5% (38) | 0 | 4% (3) | 4.5% (41) |
| Biweekly | 30% (230) | 30% (14) | 34% (29) | 30% (273) |
| Monthly | 16% (125) | 33% (15) | 20% (17) | 17% (157) |
| Bimonthly | 5% (42) | 19% (9) | 6% (5) | 6% (56) |
| When necessary | 1% (8) | 2% (1) | 1% (1) | 1% (10) |
| Just replenish the water | 3% (22) | 0 | 0 | 2.5% (22) |
| Other answers | 2% (15) | 2% (1) | 1% (1) | 2% (17) |
| Have any of your fish ever gotten sick? |  |  |  |  |
| No | 26% (205) | 28% (13) | 15% (13) | 25% (231) |
| Yes | 73% (571) | 72% (33) | 84% (71) | 75% (675) |
| Where did you seek treatment? |  |  |  |  |
| With others aquarists | 40% (349) | 42% (23) | 38% (47) | 40% (419) |
| On the Internet | 38% (329) | 33% (18) | 34% (42) | 37% (389) |
| With professionals | 21% (182) | 24% (13) | 27% (34) | 22% (229) |
|  |  |  |  |  |

**Table 4A.** Freshwater ornamental fish species kept by Brazilian fishkeepers. Conservation status of freshwater fish species kept by Brazilian fishkeepers interviewed: IUCN: NE = Not Evaluated; DD = Data Deficient; LC = Least Concern; NT = Near threatened; VU = Vulnerable; EN = Endangered; CR = Critically endangered. CITES: (I) Appendix I – species under serious threat of extinction; (II) Appendix II – species vulnerable to exploitation whose trade must be controlled; (III) Appendix III – species protected in at least one country.

| **Family** | **Species** | **Citations (n)** | **Native** | **IUCN** | **CITES** | **MMA/445** | **Brazilian legislation** |
| --- | --- | --- | --- | --- | --- | --- | --- |
| Ambassidae | *Parambassis ranga* | 3 | No | LC | - | - | Allowed |
| Anabantidae | *Ctenopoma acutirostre* | 2 | No | LC | - | - | Allowed |
| Anostomidae | *Abramites hypselonotus* | 1 | Yes | NE | - | - | Allowed |
| Anostomidae | *Leporinus friderici* | 1 | Yes | NE | - | - | Forbidden |
| Anostomidae | *Leporinus macrocephalus* | 1 | Yes | NE | - | - | Forbidden |
| Anostomidae | *Megaleporinus obtusidens* | 1 | Yes | LC | - | - | Forbidden |
| Aplocheilidae | *Aplocheilus lineatus* | 2 | No | LC | - | - | Allowed |
| Apteronotidae | *Apteronotus albifrons* | 5 | No | NE | - | - | Allowed |
| Arapaimidae | *Arapaima gigas* | 10 | Yes | DD | II | - | Forbidden |
| Ariidae | *Ariopsis seemanni* | 1 | No | NE | - | - | Forbidden |
| Aspredinidae | *Bunocephalus coracoideus* | 5 | Yes | LC | - | - | Allowed |
| Auchenipteridae | *Liosomadoras oncinus* | 3 | Yes | LC | - | - | Allowed |
| Auchenipteridae | *Liosomadoras sp.* | 1 | - | - | - | - | - |
| Auchenipteridae | *Trachelyopterus sp.* | 1 | - | - | - | - | - |
| Bagridae | *Hemibagrus wyckii* | 1 | No | LC | - | - | Allowed |
| Bagridae | *Hyalobagrus flavus* | 1 | No | NE | - | - | Forbidden |
| Balitoridae | *Pseudogastromyzon myersi* | 1 | No | LC | - | - | Allowed |
| Bryconidae | *Brycon cephalus* | 3 | Yes | NE | - | - | Forbidden |
| Bryconidae | *Brycon hilarii* | 2 | No | NE | - | - | Forbidden |
| Bryconidae | *Brycon orbignyanus* | 1 | Yes | NE | - | - | Forbidden |
| Bryconidae | *Salminus brasiliensis* | 3 | Yes | NE | - | - | Forbidden |
| Bryconidae | *Salminus hilarii* | 1 | Yes | NE | - | - | Forbidden |
| Callichthyidae | *Callichthys callichthys* | 1 | No | NE | - | - | Allowed |
| Callichthyidae | *Corydoras adolfoi* | 2 | Yes | LC | - | - | Allowed |
| Callichthyidae | *Corydoras aeneus* | 9 | No | NE | - | - | Allowed |
| Callichthyidae | *Corydoras elegans* | 2 | Yes | NE | - | - | Allowed |
| Callichthyidae | *Corydoras hastatus* | 7 | Yes | NE | - | - | Allowed |
| Callichthyidae | *Corydoras julii* | 1 | Yes | NE | - | - | Allowed |
| Callichthyidae | *Corydoras melini* | 1 | Yes | LC | - | - | Allowed |
| Callichthyidae | *Corydoras paleatus* | 3 | Yes | NE | - | - | Allowed |
| Callichthyidae | *Corydoras panda* | 1 | Yes | NT | - | - | Allowed |
| Callichthyidae | *Corydoras schwartzi* | 9 | Yes | NE | - | - | Allowed |
| Callichthyidae | *Corydoras sp.* | 173 | - | - | - | - | - |
| Callichthyidae | *Corydoras sterbai* | 2 | Yes | NE | - | - | Allowed |
| Callichthyidae | *Dianema urostriatum* | 1 | Yes | NE | - | - | Allowed |
| Callichthyidae | *Hoplosternum littorale* | 14 | Yes | NE | - | - | Allowed |
| Callichthyidae | *Megalechis thoracata* | 1 | Yes | NE | - | - | Allowed |
| Centrarchidae | *Micropterus salmoides* | 1 | No | LC | - | - | Forbidden |
| Channidae | *Channa aurantimaculata* | 1 | No | DD | - | - | Forbidden |
| Channidae | *Channa marulioides* | 1 | No | LC | - | - | Forbidden |
| Characidae | *Aphyocharax anisitsi* | 5 | No | NE | - | - | Allowed |
| Characidae | *Astyanax bimaculatus* | 10 | Yes | NE | - | - | Allowed |
| Characidae | *Astyanax mexicanus* | 1 | No | LC | - | - | Allowed |
| Characidae | *Astyanax sp.* | 1 | - | - | - | - | - |
| Characidae | *Brittanichthys axelrodi* | 1 | Yes | NE | - | - | Allowed |
| Characidae | *Chalceus erythrurus* | 1 | Yes | NE | - | - | Allowed |
| Characidae | *Galeocharax knerii* | 1 | Yes | NE | - | - | Forbidden |
| Characidae | *Gymnocorymbus ternetzi* | 58 | No | NE | - | - | Allowed |
| Characidae | *Hasemania nana* | 1 | Yes | NE | - | - | Allowed |
| Characidae | *Hemigrammus bleheri* | 1 | Yes | NE | - | - | Allowed |
| Characidae | *Hemigrammus rhodostomus* | 47 | Yes | NE | - | - | Allowed |
| Characidae | *Hemigrammus rodwayi* | 2 | No | NE | - | - | Allowed |
| Characidae | *Hemigrammus ulreyi* | 1 | Yes | NE | - | - | Allowed |
| Characidae | *Hyphessobrycon amandae* | 20 | No | NE | - | - | Allowed |
| Characidae | *Hyphessobrycon anisitsi* | 8 | No | NE | - | - | Allowed |
| Characidae | *Hyphessobrycon elachys* | 2 | Yes | NE | - | - | Allowed |
| Characidae | *Hyphessobrycon eques* | 138 | Yes | NE | - | - | Allowed |
| Characidae | *Hyphessobrycon erythrostigma* | 6 | Yes | NE | - | - | Allowed |
| Characidae | *Hyphessobrycon flammeus* | 9 | Yes | NE | - | - | Forbidden |
| Characidae | *Hyphessobrycon herbertaxelrodi* | 10 | No | NE | - | - | Allowed |
| Characidae | *Hyphessobrycon megalopterus* | 7 | Yes | LC | - | - | Allowed |
| Characidae | *Hyphessobrycon pulchripinnis* | 8 | Yes | LC | - | - | Allowed |
| Characidae | *Hyphessobrycon sweglesi* | 2 | No | NE | - | - | Allowed |
| Characidae | *Hyphessobrycon takasei* | 1 | Yes | NE | - | - | Allowed |
| Characidae | *Hyphessobrycon vilmae* | 1 | Yes | NE | - | - | Allowed |
| Characidae | *Hypressobrycon sp.* | 1 | - | - | - | - | - |
| Characidae | *Inpaichthys kerri* | 2 | Yes | NE | - | - | Allowed |
| Characidae | *Moenkhausia costae* | 15 | Yes | NE | - | - | Allowed |
| Characidae | *Moenkhausia sanctaefilomenae* | 3 | Yes | NE | - | - | Allowed |
| Characidae | *Paracheirodon axelrodi* | 54 | Yes | NE | - | - | Allowed |
| Characidae | *Paracheirodon innesi* | 131 | Yes | NE | - | - | Allowed |
| Characidae | *Pristella maxillaris* | 2 | Yes | NE | - | - | Allowed |
| Characidae | *Thayeria boehlkei* | 1 | Yes | NE | - | - | Allowed |
| Characidae | *Thayeria obliqua* | 1 | Yes | NE | - | - | Allowed |
| Characidae | *Tucanoichthys tucano* | 1 | Yes | NE | - | - | Allowed |
| Chilodontidae | *Chilodus punctatus* | 1 | Yes | NE | - | - | Allowed |
| Cichlidae | *Acarichthys heckelii* | 1 | Yes | NE | - | - | Allowed |
| Cichlidae | *Altolamprologus calvus* | 7 | No | NT | - | - | Allowed |
| Cichlidae | *Altolamprologus compressiceps* | 4 | No | LC | - | - | Allowed |
| Cichlidae | *Amatitlania nigrofasciata* | 22 | No | NE | - | - | Forbidden |
| Cichlidae | *Amphilophus citrinellus* | 69 | No | NE | - | - | Allowed |
| Cichlidae | *Amphilophus citrinellus x Paraneetroplus synspilus* | 53 | No | NE | - | - | Forbidden |
| Cichlidae | *Amphilophus labiatus* | 18 | No | NE | - | - | Allowed |
| Cichlidae | *Andinoacara pulcher* | 9 | No | NE | - | - | Forbidden |
| Cichlidae | *Andinoacara rivulatus* | 33 | No | NE | - | - | Allowed |
| Cichlidae | *Apistogramma agassizii* | 3 | Yes | NE | - | - | Allowed |
| Cichlidae | *Apistogramma bitaeniata* | 1 | Yes | NE | - | - | Allowed |
| Cichlidae | *Apistogramma borellii* | 3 | No | NE | - | - | Allowed |
| Cichlidae | *Apistogramma cacatuoides* | 13 | No | NE | - | - | Allowed |
| Cichlidae | *Apistogramma paucisquamis* | 1 | Yes | NE | - | - | Allowed |
| Cichlidae | *Apistogramma uaupesi* | 1 | Yes | NE | - | - | Allowed |
| Cichlidae | *Aristochromis christyi* | 2 | No | LC | - | - | Allowed |
| Cichlidae | *Astronotus ocellatus* | 157 | Yes | NE | - | - | Allowed |
| Cichlidae | *Aulonocara baenschi* | 3 | No | CR | - | - | Allowed |
| Cichlidae | *Aulonocara hansbaenschi* | 1 | No | VU | - | - | Allowed |
| Cichlidae | *Aulonocara jacobfreibergi* | 41 | No | LC | - | - | Allowed |
| Cichlidae | *Aulonocara maylandi* | 14 | No | CR | - | - | Allowed |
| Cichlidae | *Aulonocara nyassae* | 12 | No | NT | - | - | Allowed |
| Cichlidae | *Aulonocara sp.* | 3 | - | - | - | - | - |
| Cichlidae | *Aulonocara steveni* | 4 | No | VU | - | - | Forbidden |
| Cichlidae | *Aulonocara stuartgranti* | 43 | No | LC | - | - | Allowed |
| Cichlidae | *Australoheros facetus* | 2 | Yes | NE | - | - | Forbidden |
| Cichlidae | *Buccochromis lepturus* | 1 | No | LC | - | - | Forbidden |
| Cichlidae | *Champsochromis caeruleus* | 1 | No | LC | - | - | Forbidden |
| Cichlidae | *Champsochromis spilorhynchus* | 1 | No | EN | - | - | Forbidden |
| Cichlidae | *Chindongo elongatus* | 5 | No | NT | - | - | Allowed |
| Cichlidae | *Chindongo saulosi* | 2 | No | CR | - | - | Forbidden |
| Cichlidae | *Cichla kelberi* | 10 | Yes | NE | - | - | Forbidden |
| Cichlidae | *Cichla melaniae* | 1 | Yes | NE | - | - | Forbidden |
| Cichlidae | *Cichla monoculus* | 1 | Yes | NE | - | - | Forbidden |
| Cichlidae | *Cichla ocellaris* | 19 | No | NE | - | - | Forbidden |
| Cichlidae | *Cichla orinocensis* | 1 | Yes | NE | - | - | Forbidden |
| Cichlidae | *Cichla pinima* | 3 | Yes | NE | - | - | Forbidden |
| Cichlidae | *Cichla piquiti* | 6 | Yes | NE | - | - | Forbidden |
| Cichlidae | *Cichla sp.* | 5 | - | - | - | - | - |
| Cichlidae | *Cichlasoma paranaense* | 1 | Yes | NE | - | - | Forbidden |
| Cichlidae | *Cichlasoma portalegrense* | 1 | Yes | NE | - | - | Allowed |
| Cichlidae | *Cichlasoma trimaculatum* | 2 | No | NE | - | - | Allowed |
| Cichlidae | *Copadichromis borleyi* | 6 | No | LC | - | - | Allowed |
| Cichlidae | *Coptodon rendalli* | 2 | No | LC | - | - | Forbidden |
| Cichlidae | *Crenicichla lacustris* | 10 | Yes | NE | - | - | Forbidden |
| Cichlidae | *Crenicichla lenticulata* | 1 | Yes | NE | - | - | Allowed |
| Cichlidae | *Crenicichla lepidota* | 2 | Yes | LC | - | - | Allowed |
| Cichlidae | *Cynotilapia afra* | 10 | No | LC | - | - | Allowed |
| Cichlidae | *Cyphotilapia frontosa* | 9 | No | LC | - | - | Allowed |
| Cichlidae | *Cyrtocara moorii* | 5 | No | VU | - | - | Allowed |
| Cichlidae | *Dicrossus filamentosus* | 1 | No | NE | - | - | Allowed |
| Cichlidae | *Dimidiochromis compressiceps* | 5 | No | LC | - | - | Allowed |
| Cichlidae | *Etroplus maculatus* | 8 | No | LC | - | - | Allowed |
| Cichlidae | *Flowerhorn* | 23 | No | NE | - | - | Forbidden |
| Cichlidae | *Fossorochromis rostratus* | 3 | No | LC | - | - | Allowed |
| Cichlidae | *Geophagus brasiliensis* | 23 | Yes | NE | - | - | Allowed |
| Cichlidae | *Geophagus proximus* | 1 | Yes | NE | - | - | Allowed |
| Cichlidae | *Geophagus sveni* | 1 | Yes | NE | - | - | Forbidden |
| Cichlidae | *Gephyrochromis moorii* | 6 | No | LC | - | - | Allowed |
| Cichlidae | *Gymnogeophagus rhabdotus* | 6 | Yes | NE | - | - | Allowed |
| Cichlidae | *Haplochromis aeneocolor* | 1 | No | VU | - | - | Forbidden |
| Cichlidae | *Haplochromis latifasciatus* | 2 | No | CR | - | - | Forbidden |
| Cichlidae | *Haplochromis nyererei* | 1 | No | LC | - | - | Forbidden |
| Cichlidae | *Haplochromis obliquidens* | 1 | No | VU | - | - | Allowed |
| Cichlidae | *Hemichromis bimaculatus* | 7 | No | LC | - | - | Forbidden |
| Cichlidae | *Herichthys carpintis* | 8 | No | NE | - | - | Allowed |
| Cichlidae | *Herichthys cyanoguttatus* | 23 | No | LC | - | - | Forbidden |
| Cichlidae | *Heros efasciatus* | 5 | Yes | NE | - | - | Allowed |
| Cichlidae | *Heros severus* | 34 | Yes | NE | - | - | Allowed |
| Cichlidae | *Heterotilapia buttikoferi* | 3 | No | NE | - | - | Allowed |
| Cichlidae | *híbrido citrinellum X papagaio* | 1 | No | NE | - | - | Forbidden |
| Cichlidae | *Hoplarchus psittacus* | 17 | Yes | NE | - | - | Allowed |
| Cichlidae | *Hypselecara temporalis* | 8 | Yes | NE | - | - | Allowed |
| Cichlidae | *Julidochromis marlieri* | 3 | No | LC | - | - | Allowed |
| Cichlidae | *Julidochromis transcriptus* | 1 | No | LC | - | - | Allowed |
| Cichlidae | *Labeotropheus trewavasae* | 34 | No | LC | - | - | Allowed |
| Cichlidae | *Labidochromis caeruleus* | 44 | No | LC | - | - | Allowed |
| Cichlidae | *Laetacara curviceps* | 4 | Yes | NE | - | - | Allowed |
| Cichlidae | *Laetacara dorsigera* | 1 | Yes | NE | - | - | Allowed |
| Cichlidae | *Lamprologus ocellatus* | 3 | No | LC | - | - | Allowed |
| Cichlidae | *Maskaheros argenteus* | 9 | No | NE | - | - | Forbidden |
| Cichlidae | *Mayaheros urophthalmus* | 6 | No | NE | - | - | Forbidden |
| Cichlidae | *Maylandia estherae* | 7 | No | LC | - | - | Allowed |
| Cichlidae | *Maylandia lombardoi* | 29 | No | LC | - | - | Allowed |
| Cichlidae | *Melanochromis auratus* | 29 | No | LC | - | - | Allowed |
| Cichlidae | *Melanochromis chipokae* | 26 | No | CR | - | - | Allowed |
| Cichlidae | *Mesoheros festae* | 1 | No | NE | - | - | Allowed |
| Cichlidae | *Mesonauta festivus* | 1 | Yes | NE | - | - | Allowed |
| Cichlidae | *Mikrogeophagus altispinosus* | 13 | Yes | NE | - | - | Allowed |
| Cichlidae | *Mikrogeophagus ramirezi* | 44 | No | NE | - | - | Allowed |
| Cichlidae | *Nandopsis tetracanthus* | 1 | No | NE | - | - | Allowed |
| Cichlidae | *Neolamprologus brichardi* | 7 | No | LC | - | - | Allowed |
| Cichlidae | *Neolamprologus cylindricus* | 1 | No | NE | - | - | Allowed |
| Cichlidae | *Neolamprologus leleupi* | 8 | No | NE | - | - | Allowed |
| Cichlidae | *Neolamprologus multifasciatus* | 1 | No | LC | - | - | Allowed |
| Cichlidae | *Nimbochromis linvigstone* | 1 | No | LC | - | - | Allowed |
| Cichlidae | *Nimbochromis livingstonii* | 5 | No | LC | - | - | Allowed |
| Cichlidae | *Nimbochromis venustus* | 6 | No | LC | - | - | Allowed |
| Cichlidae | *Oreochromis niloticus* | 31 | No | LC | - | - | Forbidden |
| Cichlidae | *Parachromis managuensis* | 5 | No | NE | - | - | Forbidden |
| Cichlidae | *Paratilapia polleni* | 6 | No | VU | - | - | Forbidden |
| Cichlidae | *Pelvicachromis pulcher* | 6 | No | LC | - | - | Allowed |
| Cichlidae | *Placidochromis phenochilus* | 1 | No | EN | - | - | Allowed |
| Cichlidae | *Pseudotropheus crabro* | 1 | No | LC | - | - | Allowed |
| Cichlidae | *Pseudotropheus demasoni* | 32 | No | VU | - | - | Allowed |
| Cichlidae | *Pseudotropheus socolofi* | 29 | No | LC | - | - | Allowed |
| Cichlidae | *Pseudotropheus cyaneorhabdos* | 30 | No | CR | - | - | Forbidden |
| Cichlidae | *Pterophyllum leopoldi* | 1 | Yes | NE | - | - | Allowed |
| Cichlidae | *Pterophyllum scalare* | 199 | Yes | NE | - | - | Allowed |
| Cichlidae | *Rocio octofasciata* | 25 | No | NE | - | - | Allowed |
| Cichlidae | *Satanoperca lilith* | 1 | Yes | NE | - | - | Allowed |
| Cichlidae | *Satonoperca jurupari* | 1 | Yes | NE | - | - | Forbidden |
| Cichlidae | *Sciaenochromis ahli* | 1 | No | LC | - | - | Allowed |
| Cichlidae | *Sciaenochromis fryeri* | 4 | No | LC | - | - | Allowed |
| Cichlidae | *Symphysodon aequifasciatus* | 74 | Yes | NE | - | - | Allowed |
| Cichlidae | *Symphysodon discus* | 7 | Yes | NE | - | - | Allowed |
| Cichlidae | *Thorichthys meeki* | 4 | No | NE | - | - | Allowed |
| Cichlidae | *Trichromis salvini* | 11 | No | NE | - | - | Allowed |
| Cichlidae | *Tropheus brichardi* | 1 | No | LC | - | - | Allowed |
| Cichlidae | *Tropheus duboisi* | 2 | No | VU | - | - | Allowed |
| Cichlidae | *Tropheus moorii* | 9 | No | LC | - | - | Allowed |
| Cichlidae | *Tyrannochromis macrostoma* | 1 | No | LC | - | - | Forbidden |
| Cichlidae | *Tyrannochromis nigriventer* | 1 | No | LC | - | - | Forbidden |
| Cichlidae | *Uaru amphiacanthoides* | 11 | Yes | NE | - | - | Allowed |
| Cichlidae | *Vieja maculicauda* | 2 | No | NE | - | - | Allowed |
| Cichlidae | *Vieja melanura* | 8 | No | NE | - | - | Allowed |
| Clariidae | *Clarias gariepinus* | 2 | No | LC | - | - | Forbidden |
| Cobitidae | *Botia almorhae* | 19 | No | LC | - | - | Forbidden |
| Cobitidae | *Chromobotia macracanthus* | 24 | No | NE | - | - | Allowed |
| Cobitidae | *Misgurnus anguillicaudatus* | 18 | No | LC | - | - | Allowed |
| Cobitidae | *Pangio kuhlii* | 17 | No | NE | - | - | Allowed |
| Cobitidae | *Syncrossus hymenophysa* | 1 | No | LC | - | - | Allowed |
| Cobitidae | *Yasuhikotakia modesta* | 1 | No | LC | - | - | Allowed |
| Cobitidae | *Yasuhikotakia morleti* | 1 | No | LC | - | - | Allowed |
| Crenuchidae | *Characidium fasciatum* | 43 | Yes | NE | - | - | Allowed |
| Ctenoluciidae | *Ctenolucius hujeta* | 1 | No | NE | - | - | Allowed |
| Cynodontidae | *Hydrolycus tatauaia* | 1 | Yes | NE | - | - | Allowed |
| Cyprinidae | *Balantiocheilos melanopterus* | 5 | No | VU | - | - | Allowed |
| Cyprinidae | *Barbodes semifasciolatus* | 17 | No | LC | - | - | Forbidden |
| Cyprinidae | *Barbonymus schwanenfeldii* | 10 | No | LC | - | - | Allowed |
| Cyprinidae | *Carassius auratus* | 165 | No | NE | - | - | Allowed |
| Cyprinidae | *Crossocheilus oblongus* | 6 | No | LC | - | - | Allowed |
| Cyprinidae | *Crossocheilus siamensis* | 1 | No | LC | - | - | Allowed |
| Cyprinidae | *Ctenopharyngodon idella* | 3 | No | NE | - | - | Forbidden |
| Cyprinidae | *Cyprinus carpio* | 71 | No | VU | - | - | Allowed |
| Cyprinidae | *Danio kyathit* | 1 | No | NT | - | - | Allowed |
| Cyprinidae | *Danio margaritatus* | 7 | No | DD | - | - | Allowed |
| Cyprinidae | *Danio rerio* | 107 | No | LC | - | - | Allowed |
| Cyprinidae | *Devario malabaricus* | 4 | No | LC | - | - | Forbidden |
| Cyprinidae | *Epalzeorhynchos bicolor* | 20 | No | CR | - | - | Allowed |
| Cyprinidae | *Epalzeorhynchos frenatum* | 16 | No | LC | - | - | Allowed |
| Cyprinidae | *Epalzeorhynchos kalopterus* | 11 | No | DD | - | - | Allowed |
| Cyprinidae | *Hypophthalmichthys nobilis* | 1 | No | DD | - | - | Forbidden |
| Cyprinidae | *Labeo chrysophekadion* | 6 | No | LC | - | - | Allowed |
| Cyprinidae | *Pethia conchonius* | 10 | No | LC | - | - | Allowed |
| Cyprinidae | *Pethia nigrofasciata* | 1 | No | VU | - | - | Forbidden |
| Cyprinidae | *Pethia padamya* | 1 | No | DD | - | - | Forbidden |
| Cyprinidae | *Puntigrus tetrazona* | 37 | No | NE | - | - | Allowed |
| Cyprinidae | *Puntius arulius* | 1 | No | EN | - | - | Allowed |
| Cyprinidae | *Puntius titteya* | 8 | No | VU | - | - | Forbidden |
| Cyprinidae | *Sahyadria denisonii* | 1 | No | EN | - | - | Allowed |
| Cyprinidae | *Tanichthys albonubes* | 17 | No | DD | - | - | Allowed |
| Cyprinidae | *Trigonostigma hengeli* | 1 | No | NE | - | - | Allowed |
| Cyprinodontiformes | *Aphyosemion australe* | 7 | No | LC | - | - | Allowed |
| Datnioididae | *Datnioides polota* | 1 | No | LC | - | - | Allowed |
| Datnioididae | *Datnioides undecimradiatus* | 1 | No | VU | - | - | Forbidden |
| Doradidae | *Acanthodoras sp.* | 1 | - | - | - | - | - |
| Doradidae | *Acanthodoras spinosissimus* | 1 | Yes | NE | - | - | Allowed |
| Doradidae | *Agamyxis pectinifrons* | 2 | Yes | NE | - | - | Allowed |
| Doradidae | *Amblydoras sp.* | 1 | - | - | - | - | - |
| Doradidae | *Astrodoras asterifrons* | 1 | Yes | NE | - | - | Allowed |
| Doradidae | *Datnioides microlepis* | 11 | No | NE | - | - | Forbidden |
| Doradidae | *Hassar wilderi* | 1 | Yes | NE | - | - | Allowed |
| Doradidae | *Megalodoras uranoscopus* | 2 | Yes | NE | - | - | Allowed |
| Doradidae | *Oxydoras niger* | 1 | Yes | NE | - | - | Forbidden |
| Doradidae | *Platydoras armatulus* | 1 | Yes | NE | - | - | Allowed |
| Doradidae | *Platydoras sp.* | 1 | - | - | - | - | - |
| Doradidae | *Pterodoras granulosus* | 1 | No | NE | - | - | Forbidden |
| Doradidae | *Pterodoras sp.* | 1 | - | - | - | - | - |
| Erythrinidae | *Hoplerythrinus unitaeniatus* | 1 | Yes | NE | - | - | Forbidden |
| Erythrinidae | *Hoplias malabaricus* | 8 | Yes | NE | - | - | Allowed |
| Gasteropelecidae | *Carnegiella marthae* | 1 | Yes | NE | - | - | Allowed |
| Gasteropelecidae | *Carnegiella strigata* | 3 | Yes | NE | - | - | Allowed |
| Gobiidae | *Ctenogobius shufeldti* | 1 | Yes | LC | - | - | Forbidden |
| Gobiidae | *Periophthalmus sp.* | 1 | - | - | - | - | - |
| Gymnotidae | *Electrophorus electricus* | 1 | Yes | LC | - | - | Forbidden |
| Gymnotidae | *Gymnotus cf. carapo* | 1 | Yes | NE | - | - | Allowed |
| Gyrinocheilidae | *Gyrinocheilus aymonieri* | 27 | No | LC | - | - | Allowed |
| Helostomatidae | *Helostoma temminckii* | 9 | No | LC | - | - | Allowed |
| Hemiodontidae | *Hemiodus gracilis* | 5 | Yes | NE | - | - | Allowed |
| Heptapteridae | *Rhamdia quelen* | 20 | Yes | NE | - | - | Forbidden |
| Heptapteridae | *Rhamdioglanis transfasciatus* | 1 | Yes | NE | - | - | Forbidden |
| Lebiasinidae | *Copella nattereri* | 2 | Yes | NE | - | - | Allowed |
| Lebiasinidae | *Nannostomus anduzei* | 1 | Yes | NE | - | - | Allowed |
| Lebiasinidae | *Nannostomus beckfordi* | 2 | Yes | NE | - | - | Allowed |
| Lebiasinidae | *Nannostomus marginatus* | 2 | Yes | NE | - | - | Allowed |
| Lebiasinidae | *Nannostomus unifasciatus* | 3 | Yes | NE | - | - | Allowed |
| Lepidosirenidae | *Lepidosiren paradoxa* | 2 | Yes | NE | - | - | Forbidden |
| Lepisosteidae | *Atractosteus spatula* | 1 | No | NE | - | - | Forbidden |
| Lepisosteidae | *Lepisosteus oculatus* | 41 | No | NE | - | - | Allowed |
| Loricariidae | *Ancistrus dolichopterus* | 7 | Yes | LC | - | - | Allowed |
| Loricariidae | *Ancistrus multispinis* | 3 | Yes | NE | - | - | Allowed |
| Loricariidae | *Ancistrus ranunculus* | 2 | Yes | NE | - | - | Allowed |
| Loricariidae | *Baryancistrus sp.* | 2 | - | - | - | - | - |
| Loricariidae | *Baryancistrus sp. (L142)* | 1 | Yes | NE | - | - | Allowed |
| Loricariidae | *Baryancistrus xanthellus* | 7 | Yes | NE | - | - | Allowed |
| Loricariidae | *Dekeyseria brachyura* | 1 | Yes | NE | - | - | Allowed |
| Loricariidae | *Hemiancistrus sabaji* | 2 | Yes | NE | - | - | Allowed |
| Loricariidae | *Hypancistrus inspector* | 1 | Yes | NE | - | - | Allowed |
| Loricariidae | *Hypancistrus sp. (L066)* | 1 | Yes | NE | - | - | Allowed |
| Loricariidae | *Hypancistrus sp. (L174)* | 1 | Yes | NE | - | - | Forbidden |
| Loricariidae | *Hypancistrus sp. (L236)* | 1 | Yes | NE | - | - | Forbidden |
| Loricariidae | *Hypancistrus sp. (L260)* | 1 | Yes | NE | - | - | Allowed |
| Loricariidae | *Hypancistrus sp. (L333)* | 3 | Yes | NE | - | - | Allowed |
| Loricariidae | *Hypancistrus sp. (L400)* | 1 | Yes | NE | - | - | Forbidden |
| Loricariidae | *Hypancistrus zebra* | 4 | Yes | NE | III | CR | Forbidden |
| Loricariidae | *Hypostomus plecostomus* | 177 | No | NE | - | - | Allowed |
| Loricariidae | *Hypostomus soniae* | 2 | Yes | NE | - | - | Allowed |
| Loricariidae | *Hypostomus sp.* | 1 | - | - | - | - | - |
| Loricariidae | *Leporacanthicus joselimai* | 4 | Yes | NE | - | VU | Allowed |
| Loricariidae | *Loricaria simillima* | 1 | Yes | NE | - | - | Forbidden |
| Loricariidae | *Otocinclus affinis* | 83 | Yes | NE | - | - | Allowed |
| Loricariidae | *Panaqolus sp. (L397)* | 1 | Yes | NE | - | - | Forbidden |
| Loricariidae | *Panaque sp.* | 2 | - | - | - | - | - |
| Loricariidae | *Panaque sp. (L002)* | 2 | Yes | NE | - | - | Allowed |
| Loricariidae | *Parancistrus aurantiacus* | 1 | Yes | NE | - | - | Allowed |
| Loricariidae | *Parotocinclus jumbo* | 1 | Yes | NE | - | - | Forbidden |
| Loricariidae | *Peckoltia compta* | 1 | Yes | NE | - | EN | Allowed |
| Loricariidae | *Peckoltia vittata* | 5 | Yes | NE | - | - | Allowed |
| Loricariidae | *Pseudacanthicus leopardus* | 7 | No | NE | - | - | Allowed |
| Loricariidae | *Pseudacanthicus pirarara* | 1 | Yes | NE | - | - | Forbidden |
| Loricariidae | *Pseudacanthicus pitanga* | 1 | Yes | NE | - | - | Allowed |
| Loricariidae | *Pseudacanthicus sp.* | 1 | - | - | - | - | - |
| Loricariidae | *Pseudacanthicus spinosus* | 1 | Yes | NE | - | - | Allowed |
| Loricariidae | *Pseudorinelepis genibarbis* | 2 | Yes | NE | - | - | Allowed |
| Loricariidae | *Pterygoplichthys pardalis* | 27 | Yes | NE | - | - | Allowed |
| Loricariidae | *Pterygoplichthys parnaibae* | 2 | Yes | NE | - | - | Forbidden |
| Loricariidae | *Rineloricaria lanceolata* | 1 | Yes | NE | - | - | Allowed |
| Loricariidae | *Scobinancistrus aureatus* | 1 | Yes | NE | - | VU | Allowed |
| Loriicaridae | *Megalancistrus parananus* | 26 | Yes | NE | - | - | Forbidden |
| Mastacembelidae | *Mastacembelus erythrotaenia* | 3 | No | LC | - | - | Allowed |
| Melanotaeniidae | *Glossolepis incisus* | 1 | No | EN | - | - | Allowed |
| Melanotaeniidae | *Melanotaenia boesemani* | 3 | No | EN | - | - | Allowed |
| Melanotaeniidae | *Melanotaenia praecox* | 2 | No | DD | - | - | Allowed |
| Mochokidae | *Synodontis eupterus* | 6 | No | LC | - | - | Allowed |
| Mochokidae | *Synodontis nigriventris* | 5 | No | NE | - | - | Allowed |
| Mochokidae | *Synodontis petricola* | 13 | No | LC | - | - | Allowed |
| Nandidae | *Nandus nandus* | 1 | No | LC | - | - | Allowed |
| Nemacheilidae | *Yunnanilus cruciatus* | 1 | No | LC | - | - | Forbidden |
| Nothobranchiidae | *Aphyosemion bivittatum* | 1 | No | LC | - | - | Forbidden |
| Nothobranchiidae | *Aphyosemion ogoense* | 2 | No | NE | - | - | Forbidden |
| Nothobranchiidae | *Epiplatys dagetti* | 1 | No | LC | - | - | Allowed |
| Nothobranchiidae | *Fundolupanchax sjoestedti* | 1 | No | LC | - | - | Forbidden |
| Nothobranchiidae | *Fundulopanchax gardneri* | 4 | No | LC | - | - | Forbidden |
| Nothobranchiidae | *Nothobranchius guenteri* | 1 | No | LC | - | - | Forbidden |
| Notopteridae | *Chitala blanci* | 2 | No | NT | - | - | Allowed |
| Notopteridae | *Chitala chitala* | 3 | No | NT | - | - | Allowed |
| Notopteridae | *Chitala ornata* | 26 | No | LC | - | - | Allowed |
| Osphronemidae | *Betta splendens* | 156 | No | NE | - | - | Allowed |
| Osphronemidae | *Macropodus opercularis* | 3 | No | LC | - | - | Allowed |
| Osphronemidae | *Osphronemus goramy* | 17 | No | LC | - | - | Forbidden |
| Osphronemidae | *Trichogaster chuna* | 1 | No | LC | - | - | Allowed |
| Osphronemidae | *Trichogaster fasciata* | 1 | No | LC | - | - | Forbidden |
| Osphronemidae | *Trichogaster labiosa* | 1 | No | LC | - | - | Forbidden |
| Osphronemidae | *Trichogaster lalius* | 50 | No | LC | - | - | Allowed |
| Osphronemidae | *Trichopodus leerii* | 2 | No | NT | - | - | Forbidden |
| Osphronemidae | *Trichopodus trichopterus* | 20 | No | LC | - | - | Forbidden |
| Osteoglossidae | *Osteoglossum bicirrhosum* | 38 | Yes | NE | - | - | Allowed |
| Osteoglossidae | *Scleropages jardinii* | 1 | No | NE | - | - | Allowed |
| Pangasiidae | *Pangasianodon hypophthalmus* | 48 | No | EN | - | - | Forbidden |
| Pimelodidae | *Phractocephalus hemioliopterus* | 16 | Yes | NE | - | - | Forbidden |
| Pimelodidae | *Pimelodus maculatus* | 3 | Yes | NE | - | - | Allowed |
| Pimelodidae | *Pseudoplatystoma corruscans* | 25 | Yes | NE | - | - | Forbidden |
| Pimelodidae | *Pseudoplatystoma corruscans x Pseudoplatystoma fasciatum* | 1 | Yes | NE | - | - | Forbidden |
| Pimelodidae | *Pseudoplatystoma fasciatum* | 4 | Yes | NE | - | - | Allowed |
| Pimelodidae | *Sorubim lima* | 7 | Yes | NE | - | - | Forbidden |
| Poeciliidae | *Poecilia latipinna* | 2 | No | LC | - | - | Allowed |
| Poeciliidae | *Poecilia reticulata* | 169 | Yes | NE | - | - | Allowed |
| Poeciliidae | *Poecilia sp.* | 1 | - | - | - | - | - |
| Poeciliidae | *Poecilia sphenops* | 100 | Yes | NE | - | - | Allowed |
| Poeciliidae | *Poecilia velifera* | 1 | No | NE | - | - | Allowed |
| Poeciliidae | *Poecilia vivipara* | 2 | Yes | NE | - | - | Forbidden |
| Poeciliidae | *Poecilia wingei* | 4 | No | NE | - | - | Forbidden |
| Poeciliidae | *Poropanchax normani* | 1 | No | LC | - | - | Allowed |
| Poeciliidae | *Xiphophorus hellerii* | 81 | No | NE | - | - | Allowed |
| Poeciliidae | *Xiphophorus maculatus* | 109 | No | NE | - | - | Allowed |
| Polypteridae | *Erpetoichthys calabaricus* | 2 | No | NT | - | - | Allowed |
| Polypteridae | *Polypterus ansorgii* | 1 | No | LC | - | - | Forbidden |
| Polypteridae | *Polypterus bichir* | 3 | No | LC | - | - | Forbidden |
| Polypteridae | *Polypterus delhezi* | 9 | No | LC | - | - | Allowed |
| Polypteridae | *Polypterus endlicheri* | 16 | No | NE | - | - | Forbidden |
| Polypteridae | *Polypterus ornatipinnis* | 4 | No | LC | - | - | Allowed |
| Polypteridae | *Polypterus senegalus* | 35 | No | NE | - | - | Allowed |
| Potamotrygonidae | *Potamotrygon falkneri* | 9 | Yes | DD | II | - | Forbidden |
| Potamotrygonidae | *Potamotrygon histrix* | 2 | Yes | NE | III | - | Allowed |
| Potamotrygonidae | *Potamotrygon leopoldi* | 1 | Yes | DD | III | - | Allowed |
| Potamotrygonidae | *Potamotrygon motoro* | 16 | Yes | DD | II | - | Allowed |
| Potamotrygonidae | *Potamotrygon sp.* | 2 | - | - | - | - | - |
| Prochilodontidae | *Prochilodus lineatus* | 2 | Yes | NE | - | - | Forbidden |
| Prochilodontidae | *Semaprochilodus insignis* | 3 | Yes | NE | - | - | Forbidden |
| Protopteridae | *Protopterus annectens* | 7 | No | NE | - | - | Forbidden |
| Pseudomugilidae | *Pseudomugil gertrudae* | 1 | No | NE | - | - | Allowed |
| Rhamphichthyidae | *Rhamphichthys hahni* | 1 | Yes | NE | - | - | Forbidden |
| Rivulidae | *Austrolebias nigripinnis* | 1 | No | NE | - | - | Forbidden |
| Rivulidae | *Kryptolebias ocellatus* | 1 | Yes | NE | - | - | Forbidden |
| Rivulidae | *Nematolebias catimbau* | 1 | Yes | NE | - | - | Forbidden |
| Rivulidae | *Nematolebias papilliferus* | 1 | Yes | NE | - | - | Forbidden |
| Rivulidae | *Nematolebias withei* | 1 | Yes | NE | - | - | Forbidden |
| Rivulidae | *Notholebias minimus* | 1 | Yes | VU | - | - | Forbidden |
| Rivulidae | *Notholebias* *vermiculatus* | 1 | Yes | NE | - | - | Forbidden |
| Serrasalmidae | *Metynnis maculatus* | 10 | Yes | NE | - | - | Allowed |
| Serrasalmidae | *Metynnis mola* | 1 | Yes | NE | - | - | Allowed |
| Serrasalmidae | *Myleus ternetzi* | 1 | Yes | NE | - | - | Allowed |
| Serrasalmidae | *Myloplus schomburgkii* | 1 | No | NE | - | - | Forbidden |
| Serrasalmidae | *Myloplus tiete* | 1 | Yes | NE | - | - | Forbidden |
| Serrasalmidae | *Mylossoma duriventre* | 1 | Yes | NE | - | - | Forbidden |
| Serrasalmidae | *Mylossoma sp.* | 1 | - | - | - | - | - |
| Serrasalmidae | *Piaractus brachypomus x Piaractus mesopotamicus* | 2 | Yes | NE | - | - | Forbidden |
| Serrasalmidae | *Piaractus mesopotamicus* | 14 | Yes | NE | - | - | Allowed |
| Serrasalmidae | *Pygocentrus nattereri* | 3 | Yes | NE | - | - | Allowed |
| Siluridae | *Kryptopterus bicirrhis* | 4 | No | LC | - | - | Allowed |
| Siluridae | *Kryptopterus bicirrhis* | 4 | No | LC | - | - | Allowed |
| Synbranchidae | *Synbranchus marmoratus* | 8 | Yes | NE | - | - | Forbidden |
| Tetraodontidae | *Colomesus asellus* | 1 | Yes | NE | - | - | Allowed |

**Table 5A.** Marine ornamental fish species kept by Brazilian fishkeepers. Conservation status of marine fish species kept by Brazilian fishkeepers interviewed: IUCN: NE = Not Evaluated; DD = Data Deficient; LC = Least Concern; NT = Near threatened; VU = Vulnerable; EN = Endangered; CR = Critically endangered; LR/CD = Lower Risk/Conservation Dependent. CITES: (I) Appendix I – species under serious threat of extinction; (II) Appendix II – species vulnerable to exploitation whose trade must be controlled; (III) Appendix III – species protected in at least one country.

| **Family** | **Species** | **Citations (n)** | **Native** | **IUCN** | **CITES** | **Brazilian legislation** |
| --- | --- | --- | --- | --- | --- | --- |
| Acanthuridae | *Zebrasoma flavescens* | 39 | No | LC | - | Allowed |
| Acanthuridae | *Paracanthurus hepatus* | 30 | No | LC | - | Allowed |
| Acanthuridae | *Acanthurus bahianus* | 8 | Yes | LC | - | Allowed |
| Acanthuridae | *Zebrasoma xanthurum* | 7 | No | LC | - | Allowed |
| Acanthuridae | *Acanthurus leucosternon* | 6 | No | LC | - | Allowed |
| Acanthuridae | *Zebrasoma desjardinii* | 6 | No | LC | - | Allowed |
| Acanthuridae | *Acanthurus japonicus* | 5 | No | LC | - | Allowed |
| Acanthuridae | *Naso lituratus* | 5 | No | LC | - | Allowed |
| Acanthuridae | *Zebrasoma scopas* | 3 | No | LC | - | Allowed |
| Acanthuridae | *Acanthurus coeruleus* | 3 | Yes | LC | - | Allowed |
| Acanthuridae | *Acanthurus chirurgus* | 2 | Yes | LC | - | Allowed |
| Acanthuridae | *Naso brevirostris* | 1 | No | LC | - | Allowed |
| Acanthuridae | *Acanthurus nigricauda* | 1 | No | LC | - | Allowed |
| Acanthuridae | *Ctenochaetus striatus* | 1 | No | LC | - | Allowed |
| Acanthuridae | *Ctenochaetus binotatus* | 1 | No | LC | - | Forbidden |
| Acanthuridae | *Acanthurus triostegus* | 1 | No | LC | - | Allowed |
| Acanthuridae | *Naso elegans* | 1 | No | LC | - | Allowed |
| Acanthuridae | *Acanthurus lineatus* | 1 | No | LC | - | Allowed |
| Acanthuridae | *Acanthurus sohal* | 1 | No | LC | - | Allowed |
| Acanthuridae | *Acanthurus tristis* | 1 | No | LC | - | Allowed |
| Acanthuridae | *Acanthurus pyroferus* | 1 | No | LC | - | Allowed |
| Acanthuridae | *Naso vlamingii* | 1 | No | LC | - | Allowed |
| Acanthuridae | *Ctenochaetus strigosus* | 1 | No | LC | - | Allowed |
| Acanthuridae | *Acanthurus olivaceus* | 1 | No | LC | - | Allowed |
| Apogonidae | *Sphaeramia nematoptera* | 5 | No | NE | - | Allowed |
| Apogonidae | *Pterapogon kauderni* | 4 | No | EN | - | Allowed |
| Balistidae | *Odonus niger* | 3 | No | NE | - | Allowed |
| Balistidae | *Melichthys niger* | 1 | Yes | LC | - | Allowed |
| Blenniidae | *Salarias fasciatus* | 10 | No | LC | - | Allowed |
| Blenniidae | *Ecsenius bicolor* | 4 | No | LC | - | Allowed |
| Blenniidae | *Ophioblennius trinitatis* | 1 | Yes | LC | - | Allowed |
| Blenniidae | *Meiacanthus nigrolineatus* | 1 | No | LC | - | Allowed |
| Blenniidae | *Parablennius marmoreus* | 1 | Yes | LC | - | Allowed |
| Blenniidae | *Scartella cristata* | 1 | Yes | LC | - | Allowed |
| Callionymidae | *Synchiropus splendidus* | 3 | No | NE | - | Allowed |
| Callionymidae | *Synchiropus splendidus* | 3 | No | NE | - | Allowed |
| Callionymidae | *Synchiropus ocellatus* | 1 | No | NE | - | Allowed |
| Chaetodontidae | *Chelmon rostratus* | 2 | No | LC | - | Allowed |
| Chaetodontidae | *Chaetodon lunula* | 1 | No | NE | - | Allowed |
| Chaetodontidae | *Heniochus acuminatus* | 1 | No | LC | - | Allowed |
| Chaetodontidae | *Chaetodon ocellatus* | 1 | Yes | LC | - | Allowed |
| Chaetodontidae | *Chaetodon striatus* | 1 | Yes | LC | - | Allowed |
| Cirrhitidae | *Cirrhitichthys falco* | 1 | No | LC | - | Allowed |
| Diodontidae | *Diodon hystrix* | 1 | Yes | LC | - | Allowed |
| Gobiidae | *Elacatinus oceanops* | 5 | No | LC | - | Allowed |
| Gobiidae | *Cryptocentrus cinctus* | 5 | No | NE | - | Allowed |
| Gobiidae | *Gobiodon histrio* | 3 | No | LC | - | Allowed |
| Gobiidae | *Gobiodon okinawae* | 3 | No | NE | - | Allowed |
| Gobiidae | *Elacatinus figaro* | 3 | Yes | NE | - | Forbidden |
| Gobiidae | *Amblyeleotris guttata* | 1 | No | NE | - | Allowed |
| Gobiidae | *Cryptocentrus leptocephalus* | 1 | No | NE | - | Allowed |
| Grammatidae | *Gramma loreto* | 9 | No | LC | - | Allowed |
| Haemulidae | *Anisotremus virginicus* | 1 | Yes | LC | - | Allowed |
| Hemiscylliidae | *Chiloscyllium punctatum* | 1 | No | NT | - | Allowed |
| Labridae | *Halichoeres melanurus* | 9 | No | LC | - | Allowed |
| Labridae | *Labroides dimidiatus* | 7 | No | LC | - | Allowed |
| Labridae | *Pseudocheilinus hexataenia* | 6 | No | LC | - | Allowed |
| Labridae | *Halichoeres chrysus* | 4 | No | LC | - | Allowed |
| Labridae | *Cirrhilabrus solorensis* | 3 | No | DD | - | Allowed |
| Labridae | *Halichoeres radiatus* | 3 | Yes | LC | - | Forbidden |
| Labridae | *Cirrhilabrus cyanopleura* | 2 | No | DD | - | Allowed |
| Labridae | *Bodianus rufus* | 1 | Yes | LC | - | Allowed |
| Labridae | *Bodianus pulchellus* | 1 | Yes | LC | - | Allowed |
| Labridae | *Gomphosus varius* | 1 | No | LC | - | Allowed |
| Labridae | *Halichoeres chloropterus* | 1 | No | LC | - | Allowed |
| Labridae | *Coris gaimard* | 1 | No | LC | - | Allowed |
| Labridae | *Thalassoma lunare* | 1 | No | LC | - | Allowed |
| Labridae | *Halichoeres brasiliensis* | 1 | Yes | DD | - | Allowed |
| Labridae | *Halichoeres penrosei* | 1 | Yes | LC | - | Forbidden |
| Labridae | *Halichoeres bivittatus* | 1 | Yes | LC | - | Allowed |
| Labridae | *Thalassoma noronhanum* | 1 | Yes | LC | - | Allowed |
| Labridae | *Doratonotus megalepis* | 1 | Yes | LC | - | Allowed |
| Labridae | *Xyrichtys splendens* | 1 | Yes | LC | - | Forbidden |
| Labridae | *Cirrhilabrus lubbocki* | 1 | No | LC | - | Allowed |
| Microdesmidae | *Nemateleotris decora* | 4 | No | LC | - | Allowed |
| Microdesmidae | *Nemateleotris magnifica* | 4 | No | LC | - | Allowed |
| Monacanthidae | *Acreichthys tomentosus* | 2 | No | LC | - | Forbidden |
| Monacanthidae | *Paramonacanthus japonicus* | 1 | No | LC | - | Forbidden |
| Muraenidae | *Gymnothorax miliaris* | 1 | Yes | LC | - | Forbidden |
| Ogcocephalidae | *Ogcocephalus vespertilio* | 1 | Yes | NE | - | Allowed |
| Ophichthidae | *Myrichthys ocellatus* | 1 | Yes | LC | - | Allowed |
| Pholidichthyidae | *Pholidichthys leucotaenia* | 3 | No | NE | - | Allowed |
| Pomacanthidae | *Centropyge bispinosa* | 4 | No | LC | - | Allowed |
| Pomacanthidae | *Centropyge loricula* | 4 | No | LC | - | Allowed |
| Pomacanthidae | *Pomacanthus paru* | 3 | Yes | NE | - | Allowed |
| Pomacanthidae | *Pomacanthus imperator* | 3 | No | LC | - | Allowed |
| Pomacanthidae | *Holacanthus ciliaris* | 2 | Yes | LC | - | Allowed |
| Pomacanthidae | *Centropyge aurantonotus* | 2 | Yes | LC | - | Allowed |
| Pomacanthidae | *Pomacanthus semicirculatus* | 1 | No | LC | - | Allowed |
| Pomacanthidae | *Chaetodontoplus mesoleucus* | 1 | No | LC | - | Allowed |
| Pomacanthidae | *Centropyge bicolor* | 1 | No | LC | - | Allowed |
| Pomacanthidae | *Pygoplites diacanthus* | 1 | No | LC | - | Allowed |
| Pomacanthidae | *Holacanthus tricolor* | 1 | Yes | LC | - | Allowed |
| Pomacentridae | *Amphiprion ocellaris* | 82 | No | NE | - | Allowed |
| Pomacentridae | *Chrysiptera parasema* | 10 | No | NE | - | Allowed |
| Pomacentridae | *Chrysiptera hemicyanea* | 9 | No | NE | - | Allowed |
| Pomacentridae | *Chromis viridis* | 5 | No | NE | - | Allowed |
| Pomacentridae | *Chrysiptera cyanea* | 4 | No | NE | - | Allowed |
| Pomacentridae | *Amphiprion percula* | 4 | No | LC | - | Allowed |
| Pomacentridae | *Premnas biaculeatus* | 3 | No | NE | - | Allowed |
| Pomacentridae | *Amphiprion frenatus* | 2 | No | LC | - | Allowed |
| Pomacentridae | *Amphiprion clarkii* | 1 | No | NE | - | Allowed |
| Pomacentridae | *Amphiprion sandaracinos* | 1 | No | LC | - | Allowed |
| Pomacentridae | *Pomacentrus caeruleus* | 1 | No | NE | - | Allowed |
| Pomacentridae | *Abudefduf saxatilis* | 1 | Yes | LC | - | Allowed |
| Pomacentridae | *Chromis multilineata* | 1 | Yes | LC | - | Allowed |
| Pomacentridae | *Dascyllus melanurus* | 1 | No | NE | - | Allowed |
| Pomacentridae | *Microspathodon chrysurus* | 1 | Yes | LC | - | Forbidden |
| Pseudochromidae | *Pseudochromis fridmani* | 9 | No | NE | - | Allowed |
| Pseudochromidae | *Pseudochromis aldabraensis* | 3 | No | NE | - | Allowed |
| Pseudochromidae | *Pseudochromis paccagnellae* | 3 | No | LC | - | Allowed |
| Pseudochromidae | *Pseudochromis sankeyi* | 1 | No | LC | - | Allowed |
| Scaridae | *Scarus zelindae* | 1 | Yes | DD | - | Allowed |
| Sciaenidae | *Pareques acuminatus* | 1 | Yes | LC | - | Allowed |
| Scorpaenidae | *Dendrochirus brachypterus* | 1 | No | LC | - | Allowed |
| Scorpaenidae | *Pterois volitans* | 1 | No | LC | - | Forbidden |
| Serranidae | *Pseudanthias squamipinnis* | 1 | No | LC | - | Allowed |
| Siganidae | *Siganus vulpinus* | 5 | No | LC | - | Allowed |
| Siganidae | *Siganus magnificus* | 1 | No | LC | - | Allowed |
| Syngnathidae | *Doryrhamphus dactyliophorus* | 1 | No | DD | - | Allowed |
| Tetraodontidae | *Canthigaster valentini* | 1 | No | NE | - | Allowed |
| Tetraodontidae | *Sphoeroides greeleyi* | 1 | Yes | LC | - | Allowed |
| Zenarchopteridae | *Dermogenys pusillus* | 1 | No | NE | - | Forbidden |

**Table 6A.** Invertebrates used in fishkeeping by Brazilian fishkeepers.

| **Phylum/Subphylum** | **Class** | **Family** | **Species/** **Vernacular name** | **Citations (n)** | **Native** | **IUCN** | **MMA 445** | **Exploration (law 9.605/98)** |
| --- | --- | --- | --- | --- | --- | --- | --- | --- |
| Annelida | Clitellata | Megascolecidae | *Perionyx excavatus* | 1 | No | NE |  | Forbidden |
| Annelida | Polychaeta | Not identified | Fireworm | 1 |  |  |  | Forbidden |
| Annelida | Not identified | Not identified | Spaghetti worm | 1 |  |  |  |  |
| Arthropoda | Branchiopoda | Artemiidae | Brine shrimp | 2 |  |  |  | Forbidden |
| Arthropoda | Branchiopoda | Daphniidae | Daphnia | 1 |  |  |  | Forbidden |
| Arthropoda | Branchiopoda | Artemiidae | *Artemia salina* | 1 | No | NE |  | Forbidden |
| Cnidaria | Anthozoa | Not identified | Corals | 19 |  |  |  | Forbidden |
| Cnidaria | Anthozoa | Not identified | Anemone | 14 |  |  |  | Forbidden |
| Cnidaria | Anthozoa | Actiniidae | *Entacmaea quadricolor* | 4 | No | NE |  | Forbidden |
| Cnidaria | Anthozoa | Not identified | Zoanthus | 1 |  |  |  | Forbidden |
| Cnidaria | Anthozoa | Sphenopidae | *Palythoa sp.* | 1 |  |  |  | Forbidden |
| Crustacea | Malacostraca | Atyidae | *Neocaridina sp.* | 84 |  |  |  | Allowed |
| Crustacea | Malacostraca | Diogenidae | *Clibanarius sp.* | 31 |  |  |  | Allowed |
| Crustacea | Malacostraca | Macrobrachium | *Macrobrachium jelskii* | 17 | Yes | LC |  | Allowed |
| Crustacea | Malacostraca | Hippolytidae | *Lysmata wurdemanni* | 10 | No | NE |  | Allowed |
| Crustacea | Malacostraca | Atyidae | *Neocaridina davidi* | 9 | No | NE |  | Allowed |
| Crustacea | Malacostraca | Atyidae | *Caridina pareparensis* var. *parvidentata* | 7 | No | LC |  | Allowed |
| Crustacea | Malacostraca | Not identified | Crab | 7 |  |  |  | Allowed |
| Crustacea | Malacostraca | Atyidae | *Neocaridina heteropoda* | 6 | No | DD |  | Allowed |
| Crustacea | Malacostraca | Epialtidae | *Stenorhynchus seticornis* | 6 | Yes | NE |  | Allowed |
| Crustacea | Malacostraca | Palaemonidae | *Macrobrachium carcinus* | 5 | Yes | LC |  | Allowed |
| Crustacea | Malacostraca | Atyidae | *Atya gabonensis* | 4 | Yes | LC |  | Allowed |
| Crustacea | Malacostraca | Atyidae | *Caridina cantonensis* | 1 | No | LC |  | Allowed |
| Crustacea | Malacostraca | Stenopodidae | *Stenopus hispidus* | 3 | Yes | NE |  | Allowed |
| Crustacea | Malacostraca | Not identified | Lobster | 3 |  |  |  | Allowed |
| Crustacea | Malacostraca | Ocypodidae | *Mithraculus forceps* | 3 | Yes | NE |  | Allowed |
| Crustacea | Malacostraca | Cambaridae | *Procambarus clarkii* | 2 | No | LC |  | Allowed |
| Crustacea | Malacostraca | Palaemonidae | *Palaemon elegans* | 2 | No | NE |  | Allowed |
| Crustacea | Malacostraca | Atyidae | *Potimirim potimirim* | 2 | Yes | LC |  | Allowed |
| Crustacea | Malacostraca | Cambaridae | *Procambarus alleni* | 1 | No | LC |  | Allowed |
| Crustacea | Malacostraca | Not identified | Hermit crab | 1 |  |  |  | Allowed |
| Crustacea | Malacostraca | Atyidae | *Neocaridina denticulata* | 1 | No | LC |  | Allowed |
| Crustacea | Malacostraca | Ocypodidae | *Minuca mordax* | 1 | Yes | NE |  | Allowed |
| Crustacea | Malacostraca | Sesarmidae | *Armases angustipes* | 1 | Yes | NE |  | Allowed |
| Crustacea | Malacostraca | Not identified | Crayfish | 1 |  |  |  | Allowed |
| Crustacea | Malacostraca | Hippolytidae | *Lysmata amboinensis* | 1 | No | NE |  | Allowed |
| Crustacea | Maxillopoda | Not identified | Copepod | 1 |  |  |  | Allowed |
| Crustacea | Ostracoda | Not identified | Ostracod | 1 |  |  |  | Allowed |
| Crustacea | Not identified | Not identified | Crustacean | 3 |  |  |  | Allowed |
| Echinodermata | Asteroidea | Not identified | Starfish | 9 |  |  |  | Forbidden |
| Echinodermata | Echinoidea | Not identified | Sea-urchin | 9 |  |  |  | Forbidden |
| Echinodermata | Holothuroidea | Not identified | Sea cucumber | 5 |  |  |  | Forbidden |
| Echinodermata | Ophiuroidea | Not identified | Offiuro | 9 |  |  |  | Forbidden |
| Echinodermata | Ophiuroidea | Ophiomyxidae | *Ophiarachna incrassata* | 1 | No | NE |  | Forbidden |
| Mollusca | Bivalvia | Cardiidae | *Tridacna sp.* | 1 |  |  |  | Allowed |
| Mollusca | Cephalopoda | Not identified | Octopus | 1 |  |  |  | Allowed |
| Mollusca | Gastropoda | Ampullariidae | *Pomacea sp.* | 109 |  |  |  | Allowed |
| Mollusca | Gastropoda | Not identified | Snail | 32 |  |  |  | Allowed |
| Mollusca | Gastropoda | Planorbidae | *Planorbarius corneus* | 23 | No | LC |  | Allowed |
| Mollusca | Gastropoda | Thiaridae | *Melanoides tuberculata* | 11 | No | LC |  | Allowed |
| Mollusca | Gastropoda | Turbinidae | *Turbo sp.* | 9 |  |  |  | Allowed |
| Mollusca | Gastropoda | Planorbidae | *Planorbella duryi* | 7 | No | NE |  | Allowed |
| Mollusca | Gastropoda | Ampullariidae | *Pomacea bridgesii* | 12 | No | LC |  | Allowed |
| Mollusca | Gastropoda | Turbinidae | *Turbo bruneus* | 6 | No | NE |  | Allowed |
| Mollusca | Gastropoda | Physidae | *Physa sp.* | 5 |  |  |  | Allowed |
| Mollusca | Gastropoda | Neritidae | *Neritina sp.* | 4 |  |  |  | Allowed |
| Mollusca | Gastropoda | Cerithiidae | *Cerithium atratum* | 3 | Yes | NE |  | Allowed |
| Mollusca | Gastropoda | Planorbidae | *Biomphalaria glabrata* | 2 | Yes | NE |  | Allowed |
| Mollusca | Gastropoda | Ampullariidae | *Pomacea canaliculata* | 2 | Yes | LC |  | Allowed |
| Mollusca | Gastropoda | Turbinidae | *Astrea sp.* | 2 |  |  |  | Allowed |
| Mollusca | Gastropoda | Neritidae | *Neritina natalensis* | 2 | No | NT |  | Allowed |
| Mollusca | Gastropoda | Spiraxidae | *Physella acuta* | 1 | No | LC |  | Allowed |
| Mollusca | Gastropoda | Planorbidae | *Planorbis sp.* | 1 |  |  |  | Allowed |
| Mollusca | Gastropoda | Pachychilidae | *Tylomelania gemmifera* | 1 | No | EN |  | Allowed |
| Mollusca | Gastropoda | Patellidae | Limpet | 1 |  |  |  | Allowed |
| Mollusca | Gastropoda | Not identified | Mollusc | 1 |  |  |  | Allowed |

**Table 7A.** Ornamental fish diseases reported by Brazilian fishkeepers and their respective treatments.

| **Disease** | **Description** | **Treatment** | **Citations (n)** |
| --- | --- | --- | --- |
| Hole in the head disease (HITH) | Or, Hexamitiasis, is erosion of sensory pores located in the head and lateral line caused by the presence of the protozoan parasite *Hexamita intestinalis* | Antibiotic, antimicrobial, coarse salt, anti-infective, bactericidal, increase in oxygen in the aquarium, increase in temperature in the aquarium, vermifuge, cleaning the area with hydrogen peroxide | 35 |
| Fish tuberculosis | *Mycobacterium* spp. infections | Antibiotic, bactericidal | 10 |
| Ichthyo (white spot disease) | Dermatological disease caused by the protozoan *Ichthyophthirius multifiliis* or *Cryptocaryon irritans* | Increase in aquarium temperature, coarse salt, parasiticide (specific for ich), fungicide, fmc solution (37% formaldehyde + malachite green + methylene blue), nitrifying bacteria compound, partial aquarium water change, antibiotic, solution bath copper, gentian violet, methylene blue, parasiticide, garlic feed, reduced light in the aquarium, almond leaves, formalin | 374 |
| Acidosis | Various symptoms associated with water acidity, such as: loss of scales, erosion of the gill epithelium, excess mucus, panting behavior, skin inflammation | Fungicide, water temperature rise, iodine | 1 |
| Fungus | - | Fungicide, salt bath, bactericide, gentian violet, potassium permanganate, topical antiseptic, compound of nitrifying bacteria, aquarium temperature increase, castanet leaves, methylene blue, almond leaves, antibiotic | 72 |
| Swim bladder | Swim bladder disorders | Fungicide, bactericide, methylene blue, analgesic, anti-gase, pea-based food, rock salt, laxative, tea, surgical procedure (in case of swelling) | 23 |
| Cotton disease | White patches or tufts, similar to cotton, on the body of the fish, caused by the fungus *Saprolegnia* sp. | Coarse salt, fungicide, methylene blue, bactericide, partial aquarium water change, aquarium temperature increase | 22 |
| Costiosis | Caused by parasitic protozoa, it affects the skin, causes a blurring of colors, itching, excessive mucus production and weakness | Water temperature rise, parasiticide (specific for ich) | 7 |
| Hydropsy | Caused by a bacterium, *Aeromonas punctatus*, the fish has a very swollen abdomen, stops feeding and swims in circles. | Antibiotic, bactericidal, rock salt, epsom salt, garlic and spinach feed, almond leaf | 34 |
| Neon disease | Caused by the protozoan *Pleistophora hyphessobryconis*, it causes the appearance of cysts in the intestinal wall | - | 3 |
| Oodinium (velvet disease) | Condition characterized by the appearance of a yellowish dust on the skin of the fish, caused by the protozoan *Oodinium pillularis* | Formaldehyde, copper quarantine, bactericide, potassium permanganate, salt, antibiotic, anthelmintic, antiparasitic, fungicide, parasiticide (specific for ich), aquarium temperature increase | 27 |
| Pop eye | Protruding eyes due to fluid buildup in the back, usually triggered by some infection | Antibiotic, bactericidal, fungicidal, methylene blue, antibiotic ointment, salt bath, antibacterial, hot water | 23 |
| Constipation | - | Laxative | 3 |
| Tumors | - | - | 3 |
| Ulcers, bedsores | - | Antibiotic, hydrogen peroxide, antibiotic ointment, salt bath, injectable antimicrobial | 6 |
| Anchor worm | Parasitic disease, whose parasitic agent is the arthropod *Lernaea cyprinacea* | Antibiotic, worm extraction, anthelmintic | 15 |
| Bacteria | - | Bactericidal, antibiotic, chestnut leaves, anti-infective | 21 |
| Parasites | Endoparasites and ectoparasites | Coarse salt, bactericidal, anti-infective, almond leaf, antibiotic, vermifuge, fungicide | 32 |
| Apathy | - | Bactericide | 1 |
| *Brooklynella hostilis* | Ciliated protozoan parasitizing marine fish | Parasiticide, formalin, antibiotic | 3 |
| *Camallanus* sp. | Nematode that parasitizes the rectum of fish | Antibiotic, anthelmintic | 3 |
| Gnawed fins | - | Fungicide, parasiticide (specific for ich), methylene blue, castanet leaves | 3 |
| Fin rot | Degeneration of fins | Iodine-free salt, antibiotics | 2 |
| Spironucleosis | Disease caused by the opportunistic protozoan *Spironucleus* sp. | Antibiotic, bitter salt | 3 |
| Wounds, bruises | Caused by fights | Tea tree extract, fungicide | 6 |
| Saprolegniose | Caused by the fungus *Saprolegnia ferax*, it causes the appearance of hyphae on the fins | Parasiticide (specific for ich), temperature rise | 1 |
