# Supplementary material for: Marine or freshwater: the role of ornamental fish keeper’s preferences in the conservation of aquatic organisms in Brazil
Source: PeerJ. 2022 Nov 11;10:e14387. doi: 10.7717/peerj.14387 (PMC9661971; doi:10.7717/peerj.14387)
Supplement: Supplemental Information 3 [file peerj-10-14387-s003.pdf]

QUESTIONÁRIO - CRIAÇÃO DE PEIXES COMO PET

Peixes criados como Pets

1 Há quanto tempo cria peixes ornamentais?
2 O que te motivou a escolher os peixes como animais de estimação?
3 Quantos peixes você tem atualmente?
4 Sabe de quantas espécies são?
5 Sabe quais são as espécies?

Questionário aplicado para cada espécie de peixe que o criador mantém em casa.

1. Qual o nome popular do peixe?
2. Sabe o nome científico? Se sim, qual é?
3. Espécie nativa do Brasil?
   a) Sim
   b) Não
4. Há quanto tempo cria o animal?
5. Quantos exemplares cria?
6. Jovem ou adulto?
7. Como você o adquiriu?

   a) Caso tenha obtido através da compra, por favor, responda as perguntas a seguir: Onde comprou? Qual foi o preço? Como ocorre a comercialização? Teve nota fiscal?

   b) Caso tenha obtido através de captura, por favor, responda as perguntas a seguir: Quem capturou? Quais os apetrechos (instrumentos) utilizados na captura? Onde o animal foi capturado? Você sabe a cidade onde o animal foi capturado (se sim, qual?)?

8. Por que escolheu essa espécie?
9. Pretende criar mais algum peixe? Se sim, qual espécie?
10. Que tipo de aquário/tanque o animal é mantido?
11. Quantas e quais espécies são criadas por aquário/tanque?
12. Qual tamanho (comprimento X altura X largura) ou volume? de cada recinto utilizado?
13. O recinto onde o animal é criado é decorado? Sem sim, por favor, responda com o que.
14. Qual é a alimentação oferecida ao animal e com frequência?
15. Como é e qual periodicidade do cuidado com o animal? (Como se dá limpeza do recinto?)
16. TPA – trocas parciais de água: periodicidade
17. Você já conseguiu reproduzi-lo? Caso sim: Quantas vezes? Quantos filhotes? O que você fez com os filhotes?
18. O animal já ficou doente? Que tipo de doença o acometeu? Qual foi o tratamento?
19. Quanto, em média, é o gasto mensal com o hobby/manutenção do aquário?

20. Se possível, me envie fotos do animal, por favor.

21. Caso você tivesse a opção de adquirir animais oriundos de extrativismo sustentável, você iria preferir essa opção?

22. Quando você quer se desfazer de algum animal que destino você dá para ele?

23. Você tem a prática de soltar peixes na natureza?

24. De onde vem a experiência em aquarismo?

a) Fóruns de aquarismo
b) Cursos
c) Buscas na internet
d) Assessoria externa?
e) Tentativa e erro?
f) Consulta a livros e literatura específica?
g) Consulta a sites especializados?

Perfil socioeconômico

Cidade: ____

6 Sexo:

a) Feminino
b) Masculino

7 Idade

a) Menos de 20
b) 20-30
c) 30-40
d) 40-50
e) Mais de 50

8 Profissão:

9 Estado civil:

a) Solteiro
b) Casado
c) Divorciado
d) Viúvo
e) União estável

10 Grau de instrução:

a) Ensino fundamental incompleto
b) Ensino fundamental completo
c) Ensino médio incompleto
d) Ensino médio completo
e) Ensino superior incompleto
f) Ensino superior completo
g) Pós-graduação

11 Qual a sua renda mensal?

a) Menor que um salário mínimo
b) Até um salário mínimo
c) Entre 1 e 2 salários mínimos

d) Entre 3 e 4 salários mínimos
e) Mais que 4 salários mínimos
